# Efficacy of transcranial magnetic stimulation and fluoxetine in the treatment of postpartum depression: A protocol for systematic review and meta-analysis

**DOI:** 10.1097/MD.0000000000020170

**Published:** 2020-05-22

**Authors:** Yan-Jun Guo, Yong-Ming Shan, Zhi-Jian Wang, Zhong-Fei Shen

**Affiliations:** a School of Medicine; b School of Mathematics and Information Engineering, Jiaxing University, Jiaxing, China.

**Keywords:** efficacy, fluoxetine, postpartum depression, safety, transcranial magnetic stimulation

## Abstract

**Background:**

Numerous studies have reported that transcranial magnetic stimulation (TMS) and fluoxetine is used in the treatment of postpartum depression (PPD). Currently, no study has systematically investigated the efficacy and safety of TMS and fluoxetine for the treatment of patients with PPD. Thus, this study will assess the efficacy and safety of TMS and fluoxetine for treating PPD.

**Methods:**

Relevant studies involving TMS and fluoxetine for the treatment of patients with PPD will be comprehensively searched from the electronic databases from inception to the February 1, 2020: Cochrane Library, EMBASE, MEDILINE, CINAHL, AMED, WANGFANG, VIP, and CNKI databases. No language and publication time restrictions will be applied. RevMan 5.3 software will be utilized for data pooling, data analysis, and risk of bias evaluation. If necessary, we will also assess reporting bias using funnel plot and Egger test.

**Results:**

This study will comprehensively summarize the existing evidence to assess the efficacy and safety of TMS and fluoxetine for treating PPD.

**Conclusion:**

The findings of this study may help to establish a better approach to treat PPD using TMS and fluoxetine.

**Dissemination and ethics:**

This study will be disseminated through a peer-reviewed journal. This study does not need ethical approval as no primary patient data will be used.

**Systematic review registration:**

INPLASY202040017.

## 1 Introduction

Postpartum depression (PPD) in women is a major depressive but treatable maternal mental disorder.^[1–4]^ It has been reported that about 10% of pregnant females and 13% of females who have just given birth experience PPD.^[5–9]^ If it cannot be treated fairly well, such condition is often associated with a high risk of stressful life events, prenatal anxiety, poor marital relationships, poor child development, and even suicide.^[10–14]^

A numerous managements have reported to treat PPD, including cognitive behavioral therapy, psychoeducaiton, psychoetherapy, acupuncture, Chinese herbal medicine, and exercise.^[1,15–21]^ However, all of them have limited efficacy. Previous studies have reported that transcranial magnetic stimulation (TMS) and fluoxetine can effectively treat PPD.^[22–30]^ However, no systematic review has addressed this issue. Thus, this study will systematically assess the efficacy and safety of TMS and fluoxetine for the treatment of patients with PPD.

## 2 Methods

### 2.1 Objective

This study aims to assess the evidence from all available randomized controlled trials (RCTs) that evaluate the combination of TMS and fluoxetine on patients with PPD.

### 2.2 Study registration

This protocol has been registered on INPLASY202040017. We have reported it based on the Cochrane Handbook for Systematic Reviews of Interventions and the Preferred Reporting Items for Systematic Reviews and Meta-Analysis Protocol statement guidelines.^[31]^

### 2.3 Inclusion criteria for study selection

#### 2.3.1 Type of studies

This study will include RCTs that compared the combination of TMS and fluoxetine with other conservative treatments. However, we will exclude any other studies, such as animal studies, case report, case series, reviews, uncontrolled trials, non-RCTs and quasi-RCTs.

#### 2.3.2 Type of participants

All adult female participants (over 18 years old) who were diagnosed as PPD will be included, regardless their country, race, educational background and economic status. However, we will exclude subjects if they had depression before the delivery.

#### 2.3.3 Type of interventions

In the experimental group, all patients must receive any types of TMS combined fluoxetine as their solely treatment.

In the control group, all participants could undergo any therapies to manage their PPD condition. However, any combinations of TMS and fluoxetine with other treatments will be excluded.

#### 2.3.4 Type of outcome measurements

The primary outcome is depression, which is measured by Hamilton Depression Scale, or Edinburgh Postpartum Depression Scale, or any other relevant scales.

The secondary outcomes are anxiety (as assessed by Hamilton Depression Scale or other associated scales); overall clinical efficacy (as reported in the trials); levels of estradiol, serotonin, adrenocorticotropic hormone, adrenocorticotropic hormone and cortisol in serum (as measured by radioimmunoassay), and adverse events.

### 2.4 Search methods for the identification of studies

#### 2.4.1 Electronic searches

We will identify relevant RCTs involving the combination of TMS and fluoxetine on patients with PPD in the electronic databases from inception to the February 1, 2020: Cochrane Library, EMBASE, MEDILINE, CINAHL, AMED, WANGFANG, VIP, and CNKI databases. We will not utilize any language and publication time restrictions to any literature search. The detailed search strategy of Cochrane Library is created as an example (Table 1). We will also adapt similar search strategies for other electronic databases.

**Table 1:**
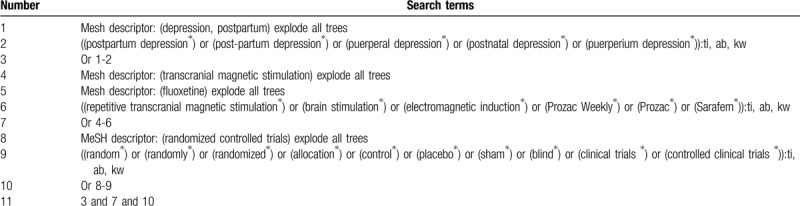
Search strategy for Cochrane Library.

#### 2.4.2 Search for other resources

Besides the above electronic databases, we will also search any relevant proceedings of conference/meeting/symposium, websites of clinical trial registry, and reference lists of related reviews.

### 2.5 Data collection and analysis

#### 2.5.1 Study selection

Two authors will independently examine the titles/abstracts of searched potential literatures based on the predefined eligibility criteria. All irrelevant literatures will be removed after the titles/abstracts screening. Then, we will investigate the full papers of all remaining trials to further test if they fulfill all inclusion criteria. We will record all excluded studies with detailed reasons and will list them in the table. Any conflicts regarding the study selection between 2 authors will be solved by a third author through discussion. The whole process of study selection will be demonstrated in a flow chart with details.

#### 2.5.2 Data extraction and management

Two authors will independently extract the associated data from each eligible trial using previously designed data collection sheet. It comprises of title, first author, publication time, location, patient characteristics, diagnostic criteria, eligibility criteria, study design, study methods, sample size, specifics of intervention and control, following up information, outcome measurements, safety, results, findings, and funding information. Any uncertainty will be solved by a third author through discussion.

#### 2.5.3 Study quality assessment

Two authors will appraise study quality of each included trial using Cochrane risk of bias tool independently. It covers 7 items, and each one is rated as “high risk of bias,” “unclear risk of bias,” and “low risk of bias.” Any controversy between 2 authors will be worked out with the help of a third author.

#### 2.5.4 Measurement of treatment effect

Considering the characteristics of the data collected from this study, all continuous data will be calculated as mean difference or standardized mean difference and 95% confidence intervals. All dichotomous data will be presented as the risk ratio and 95% confidence intervals.

#### 2.5.5 Dealing with missing data

If we identify any insufficient, unclear or even missing data, we will connect corresponding authors or relevant authors of primary trials to obtain such data. If that data is not available, we will analyze available data and will discuss its affects as a limitation.

#### 2.5.6 Assessment of heterogeneity

Statistical heterogeneity across eligible trials will be explored using *I*^2^ test. *I*^2^ ≤ 50% (the cut-off point for the present *I*^2^ statistics) represents homogeneity, and a fixed-effects model will be used. Otherwise, *I*^2^ > 50% means considerable heterogeneity, and a random-effects model will be utilized.

#### 2.5.7 Data synthesis

RevMan 5.3 software will be established for statistical analysis. If sufficient trials are included and homogeneity is identified across these trials, we will conduct a meta-analysis in according with the minor variations in study characteristics, similar interventions and comparators, and outcome measurements. If considerable heterogeneity is found among trials, we will carry out subgroup analysis to identify the sources of the obvious heterogeneity. If there is still substantial heterogeneity after subgroup analysis, a meta-analysis is deemed not be performed, and we will synthesize the outcome data using a narrative summary.

#### 2.5.8 Publication biases

If more than 10 eligible trials are included in this study, we will explore its publication bias using funnel plot,^[32]^ and Egger regression will be used to detect the funnel plot asymmetry.^[33]^

#### 2.5.9 Subgroup analysis

If necessary, subgroup analysis will be performed to explore the sources of considerable heterogeneity according to the variations in study characteristics, different types of interventions, comparators, and outcome measurements.

#### 2.5.10 Sensitivity analysis

Where appropriate, we will carry out sensitivity analysis to investigate the robustness of the study findings by excluding low quality studies.

## 3 Discussion

Previous studies have found positive efficacy of the combination of TMS and fluoxetine for the treatment of patients with PPD, and can help enhancing PPD severity. However, its efficacy and safety still remain unknown on the literature level. In addition, there is still insufficient evidence focusing on this topic. The purpose of this study is to systematically investigate the efficacy and safety of the combination of TMS and fluoxetine for the treatment of PPD. This study will be the first study that systematically evaluates the efficacy and safety of the combination of TMS and fluoxetine for the treatment of PPD in the postpartum individuals. The results of this study may help to present a better approach and to provide reliable evidence for the treatment of PPD using TMS and fluoxetine.

## Author contributions

**Conceptualization:** Yan-Jun Guo, Yong-Ming Shan, Zhi-Jian Wang, Zhong-Fei Shen.

**Data curation:** Yan-Jun Guo, Yong-Ming Shan, Zhi-Jian Wang, Zhong-Fei Shen.

**Formal analysis:** Yan-Jun Guo, Yong-Ming Shan, Zhi-Jian Wang.

**Funding acquisition:** Zhong-Fei Shen.

**Investigation:** Yan-Jun Guo, Yong-Ming Shan, Zhong-Fei Shen.

**Methodology:** Zhi-Jian Wang.

**Project administration:** Zhong-Fei Shen.

**Resources:** Yan-Jun Guo, Yong-Ming Shan, Zhi-Jian Wang.

**Software:** Yan-Jun Guo, Yong-Ming Shan, Zhi-Jian Wang.

**Supervision:** Zhong-Fei Shen.

**Validation:** Yan-Jun Guo, Yong-Ming Shan, Zhong-Fei Shen.

**Visualization:** Yan-Jun Guo, Zhi-Jian Wang, Zhong-Fei Shen.

**Writing – original draft:** Yan-Jun Guo, Yong-Ming Shan, Zhong-Fei Shen.

**Writing – review & editing:** Yan-Jun Guo, Zhi-Jian Wang, Zhong-Fei Shen.
